# Exploring Heteroplasmic Variants in mtDNA: Insights from Single-Cell Transcriptomics

**DOI:** 10.3390/cells15151403

**Published:** 2026-08-03

**Authors:** Marco Barresi, Ivano Di Meo, Alessia Nasca, Eleonora Lamantea, Andrea Legati, Daniele Ghezzi

**Affiliations:** 1 Unit of Medical Genetics and Neurogenetics, Fondazione IRCCS Istituto Neurologico Carlo Besta, 20126 Milan, Italy; marco.barresi@istituto-besta.it (M.B.); ivano.dimeo@istituto-besta.it (I.D.M.); alessia.nasca@istituto-besta.it (A.N.); eleonora.lamantea@istituto-besta.it (E.L.); andrea.legati@istituto-besta.it (A.L.); 2 Department of Pathophysiology and Transplantation (DEPT), University of Milan, 20122 Milan, Italy

**Keywords:** heteroplasmy, mitochondrial DNA, single-cell transcriptomics, mtDNA variant

## Abstract

**Highlights:**

**What are the main findings?**

- Single-cell RNA-seq reliably estimates mtDNA heteroplasmy and may reveal a predominantly bimodal, near-homoplasmic distribution across cells, not detectable by bulk approaches.
- High heteroplasmy is associated with transcriptional remodeling, but responses are largely sample-specific.

**What are the implications of the main findings?**

- Single-cell approaches are useful to dissect genotype–phenotype relationships and uncover hidden cellular heterogeneity in mitochondrial diseases.
- The influence of nuclear background and cellular context suggests that pathogenic effects of mtDNA variants cannot be generalized, emphasizing the need for personalized and cell-resolved analyses.

**Abstract:**

Mitochondrial DNA (mtDNA) heteroplasmy, which is the coexistence of wild-type and mutant mtDNA variants within the same cell, plays a critical role in modulating cellular phenotypes, disease severity, and penetrance. Bulk RNA sequencing cannot detect cell-to-cell heteroplasmy variability, limiting our understanding of the pathological mechanisms of mtDNA variants. In this study, we leveraged single-cell RNA sequencing (scRNA-seq) combined with a robust bioinformatics pipeline to characterize mtDNA heteroplasmy. We employed four fibroblast lines from patients harboring heteroplasmic mtDNA pathogenic variants in genes encoding respiratory complex I subunits. While RNA heteroplasmy corresponded to DNA-based measurements at the bulk level, single-cell analysis uncovered a diverged distribution in three out of four lines: most cells had near-homoplasmic (wild-type or mutant) mtDNA, with few cells showing intermediate levels. Furthermore, we found that high mutation levels correlate with transcriptional profile changes, although these responses were highly sample-specific, suggesting that the nuclear background and cellular context critically influence mitochondrial dysfunction and compensatory mechanisms. Our findings highlight the potential of single-cell technologies to better understand the complex link between mtDNA genetic diversity and mitochondrial phenotypic variability and to study crucial aspects of mitochondrial biology and pathology, such as clonal dynamics, at single-cell resolution.

## 1. Introduction

The study of mitochondrial heteroplasmy, defined by the coexistence of wild-type and acquired mutant variants of mitochondrial DNA (mtDNA), is crucial for understanding significant biological processes and pathologies associated with mitochondrial damage or mtDNA dysfunction. Each cell contains hundreds to thousands of mtDNA copies, and the relative frequencies of heteroplasmic variants can vary considerably among individual cells, even within the same tissue, thereby profoundly influencing cellular phenotype, suggesting dynamic processes of acquisition and selection of mitochondrial variants [1,2,3]. It is well established that the extent of heteroplasmy modulates mitochondrial functional impairment, with effects determined by both the mutational threshold and the specific nature of the mutation. Therefore, elucidating the distribution and functional consequences of these mtDNA variants is critical for understanding the pathogenic mechanisms underlying a wide range of mitochondrial, neurodegenerative, and systemic diseases and for advancing knowledge of mitochondrial biology under physiological conditions.

Bulk RNA sequencing has long been established as the primary method for analysing mtDNA variants and assessing their effect on the transcriptional profile, which provides an average estimate of allelic composition. However, this approach does not account for the considerable variability in heteroplasmy among individual cells: the same average percentage of variant measured in bulk can arise from very different distributions at the cellular level (e.g., many cells with intermediate heteroplasmy vs. a mix of nearly homoplasmic wild-type or mutant cells) [4,5]. This level of variability is crucial for interpreting the penetrance and phenotypic variability of mitochondrial diseases, as well as for understanding processes such as clonal selection and mitochondrial genetic drift.

Single-cell technologies (scRNA-seq, scATAC-seq, and multimodal approaches) have transformed investigations into this genomic and transcriptional heterogeneity, enabling the precise characterization of mtDNA variants at single-cell resolution and rigorous assessment of their effects on cellular function [6,7]. The biological relevance of these technologies has been repeatedly demonstrated in studies on lineage tracing and investigations of mtDNA segregation dynamics, both in vitro and in vivo, across diverse cell types and disease contexts [8]. Various single-cell datasets have been used to reconstruct clonal relationships by exploiting mtDNA variants as endogenous genetic barcodes and map the distribution of heteroplasmic variants within tissues, revealing associations between specific mtDNA genotypes and defined cellular subpopulations. These studies also highlight how mtDNA mutations can modulate mitochondrial network dynamics and influence cellular gene-expression programs.

The accurate estimation of heteroplasmy at single-cell resolution remains challenging [9]. Measurements can be confounded by sequencing errors, PCR and library-preparation amplification biases, uneven coverage, and misassignment of reads derived from nuclear mitochondrial DNA segments (NUMTs). Many of these limitations have been partially mitigated by recent advances, both experimental (e.g., targeted mitochondrial enrichment of scRNA libraries and full-length single-cell protocols) and computational (dedicated mtDNA variant pipelines), which integrate targeted enrichment and robust statistical and bioinformatics frameworks to obtain heteroplasmy estimates at the cell level suitable for downstream functional inference [10,11].

In this context, our study aims to expand the current understanding of mtDNA heteroplasmy by combining scRNA-seq with an integrated bioinformatics workflow to characterize the cellular distribution of known heteroplasmic mtDNA variants at single-cell resolution in an easily accessible patient-derived cell model (fibroblast lines) and evaluate the impact of extreme variant heteroplasmic percentage on global transcriptional programs.

## 2. Materials and Methods

### 2.1. RNA-Seq Library Preparation and Sequencing

Primary skin fibroblasts from four patients carrying heteroplasmic variants primarily located in the NADH dehydrogenase (*ND*) genes—m.11777C>A (*MT-ND4*), m.13513G>A (*MT-ND5*), m.13514A>G (*MT-ND5*), and m.13063G>A (*MT-ND5*)—were used in this study.

MtDNA heteroplasmy was assessed on DNA extracted from fibroblast homogenates; PCR fragments spanning the variants to test were processed with Nextera XT DNA Library Preparation kit (Illumina, San Diego, CA, USA) and sequenced afterwards on an Illumina platform as described [12]. Cells were grown in complete medium composed of Dulbecco’s Modified Eagle Medium high glucose (4.5 g/L), supplemented with 2 mM L-Gln, 100 U/mL penicillin, 10 µg/mL streptomycin, and 10% fetal bovine serum (FBS). Each cell line was collected and resuspended in DPBS, and the cell suspensions (>90% living cells examined by count) were loaded on a Chromium Single-Cell Controller (10x Genomics). Single-cell RNA sequencing was performed using the 10x Genomics Chromium Single Cell 3′ kit v3.1 (10x Genomics, Pleasanton, CA, USA). Cells were encapsulated into Gel Bead-in-Emulsions (GEMs) together with barcoded oligonucleotides, enabling reverse transcription, cDNA synthesis, and subsequent library preparation according to the manufacturer’s protocol [13]. No DNase treatment is compatible with this protocol. The scRNA-seq libraries were sequenced on the Illumina NovaSeq 6000 platform with paired-end, dual-indexing reads. Metrics of the scRNA-seq, generated using Cell Ranger (10x Genomics), are reported in Table S1.

Bulk RNAseq was performed on RNA extracted from the same fibroblast cell lines, using the TruSeq Stranded mRNA protocol (Illumina).

### 2.2. scRNA-Seq Data Processing

Raw sequencing data for all samples were processed by a data analysis workflow implemented utilizing integrated bioinformatics pipelines within Linux-based and R environments built on cluster HPC. Cellranger (v7.1.0) is used with the GRCh38 transcriptome reference to obtain per-cell alignment BAM files and filtered 10× count matrices. Across the analyzed samples, we obtained approximately 3000 to 6500 cells per sample, with mean reads per cell ranging from 161,000 to 230,000. Mitochondrial reads pileups, per-cell allele counts, and per-cell heteroplasmy estimates (allele frequency) were obtained with MitoTrace (v. 1.0.0) [14], applied to the Cellranger BAMs files along with reference genomic sequence in GRCh38.fasta format. To limit noise from low-coverage barcodes, we excluded barcodes with fewer than 100 mitochondrial reads (min_read = 100) and considered a site covered in a cell only if local coverage was ≥2 reads.

Count matrices were converted to Seurat objects (Seurat v. 5.3.0) [15]. This step removes barcodes with fewer than 100 detected features (thereby filtering potential empty/low-quality droplets) and excludes genes detected in fewer than three cells. Allele frequencies at mtDNA positions of interest were integrated into Seurat objects metadata, and to maximize cell retention for heteroplasmy profiling, we did not apply other filters on total genes detected or mitochondrial read fraction; nevertheless, percent_mito and nFeature_RNA were computed for each cell and regressed out during scaling to mitigate technical confounding. Seurat preprocessing included normalization, identification of 2000 highly variable features, scaling while regressing nFeature_RNA and percent_mito, and PCA (npcs = 20). The number of PCs retained for downstream embeddings and clustering was determined via elbow plots and cumulative variance explained; for the example dataset, 16 PCs were used. Nonlinear embeddings (UMAP, t-SNE) and clustering (resolution = 0.3) were computed on the selected PCs.

To detect and control for transcriptional variation driven by proliferation, each cell was assigned a cell-cycle phase (G1, S, G2/M) using Seurat’s CellCycleScoring with the updated S and G2/M gene lists (cc.genes.updated.2019); S.Score, G2M.Score and discrete Phase calls were added to the object metadata and used to inspect cluster composition. Cluster annotation combined Phase proportions, inspection of cluster top markers (FindAllMarkers, only.pos = TRUE; logfc.threshold = 0.25; min.pct = 0.25), and preranked gene set enrichment analysis (fgsea) performed on genes ranked by avg_log2FC (cluster versus rest); we tested S- and G2/M-phase gene sets and report ES, normalized ES (NES), empirical *p*-values and BH-adjusted *p*-values, and extracted leadingEdge genes as drivers of enrichment.

Based on the observed cell-level distributions of heteroplasmy, we defined two extreme categories for functional comparisons: low heteroplasmy (0–10%)/near homoplasmic wt and high heteroplasmy (90–100%)/near homoplasmic mutant. These categories were stored as a metadata field (HET_category) and used as the condition variable in differential expression analyses, both for per-sample and pooled plate-level comparison. Single-cell differential expression analysis was performed with MAST statistical hurdle models (v. 1.33.0) [16]. Seurat objects were converted to SingleCellAssay objects, and counts were log-transformed as log2(counts + 1). Genes expressed in fewer than 20% of cells were excluded from MAST to reduce low-signal noise. For per-sample comparisons, we simply fitted the zlm model with only the heteroplasmy categories condition and tested the contrast high vs. low. For the pooled plate-level comparison, we fit a model that included, in addition to the heteroplasmy condition, control covariates for the sample and for cell rate detection.

Relevant terms were extracted from the MAST results: hurdle *p*-values (r(>Chisq)), coefficients (coef), and component estimates for the discrete (β_D) and continuous (β_C) parts of the model. Fold-changes were computed as exp(coef) and expressed also as log2 when reported. An operational selection threshold of Pr(>Chisq) < 0.05 was used to prioritize genes for downstream annotation; false discovery rates (Benjamini–Hochberg) were computed and reported in Supplementary Tables S1–S5. To characterize the mechanism underlying expression changes, genes were classified according to the sign and concordance of coef, β_D, and β_C into categories such as Concordant_up, Concordant_down, Discrete_only_driver, Continuous_only_driver, and Discordant_other, thereby distinguishing changes driven primarily by alterations in the fraction of expressing cells from those driven by changes in per-cell expression intensity.

Differentially expressed genes were mapped to Entrez identifiers (org.Hs.eg.db) and subjected to pathway and gene ontology enrichment analyses (clusterProfiler v.4.20.0: GO BP/CC/MF, KEGG, Reactome). Mitochondrial gene enrichment was specifically using the Human MitoCarta 3.0 gene list [17] and internal panels; the fraction of DE genes corresponding to MitoCarta genes was reported, and overlaps across samples were visualized (UpSet and Heat-tile plots).

## 3. Results

### 3.1. Mitochondrial Transcript Coverage in scRNA-Sequencing

**Figure 1:**
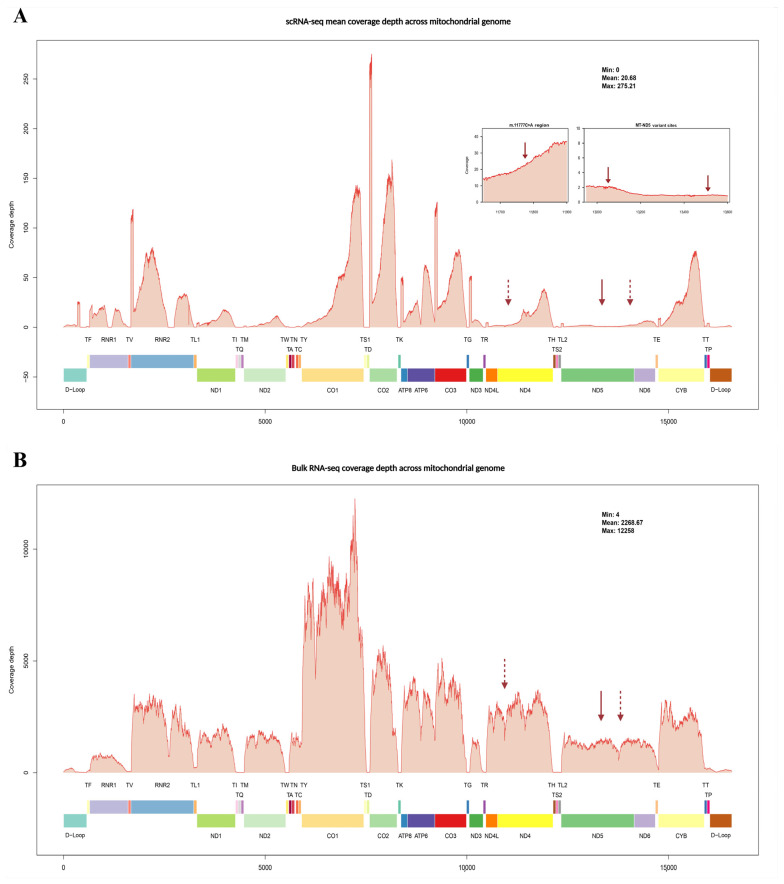
**Mitochondrial coverage profiles using scRNA-seq and bulk RNA-seq and pile-up comparison in sample S1 (m.13063G>A, *MT-ND5*).** (**A**) Mean per-cell coverage across the mitochondrial genome obtained by scRNA-seq (10× Genomics poly-A capture). The solid arrow marks the *MT-ND5* variant site in S1; dashed arrows indicate the positions of other mtDNA variants analyzed in this study. The insets show an enlarged view of the relevant low-coverage regions, highlighting the genomic region around m.11777C>A (left) and the *MT-ND5* segment (right), with arrows marking the positions of the mitochondrial variants under investigation. (**B**) Bulk RNA-seq coverage profile with a substantially higher total read count, demonstrating deep, uniform coverage of *MT-ND5* comparable to other Complex I subunits.

We focused our analysis on the mitochondrial transcriptome by extracting all reads that are mapped to the mtDNA. We evaluated the coverage of the 13 polyadenylated mRNA transcripts encoded by mtDNA and compared it with that obtained from bulk RNA-seq. The coverage was unevenly distributed and strongly biased toward the 3′ end of transcripts. In contrast, bulk RNA-seq yields deep, more uniform coverage across all mtDNA protein-coding genes (Figure 1).

### 3.2. Heteroplasmy Evaluation from scRNA-Seq Data

Since the analyzed samples harboured heteroplasmic variants in mtDNA, we assessed the mutation load in RNA-seq data. Three samples (S1–S3) had variants in *MT-ND5* and one (S4) in *MT-ND4*. Notably, both the *MT-ND5* gene (~1.8 kb) and *MT-ND4* (~1.4 kb) exhibit poor, uneven coverage in scRNA-seq data: as reported above, this may be because single-end reads generated from the 3′ terminus rarely reach their central and 5′ regions (Figure 1). Furthermore, *MT-ND5* showed the lowest mean depth of coverage amongst mitochondrial transcripts, possibly because it is one of the longest mitochondrial genes. Nonetheless, the coverage, even in these poorly represented transcripts, was sufficient for estimating RNA heteroplasmy at the single-cell level. We calculated the mean RNA heteroplasmy from pooled reads across all cells (pseudo-bulk) and compared the result with RNA mutation load detected by bulk RNA-seq or with DNA heteroplasmy. For all the variants, the mean scRNA heteroplasmy was mostly comparable to that identified in bulk RNA and DNA (Table 1).

### 3.3. Differences in RNA Heteroplasmy Among Single Cells from the Same Subject

**Figure 2:**
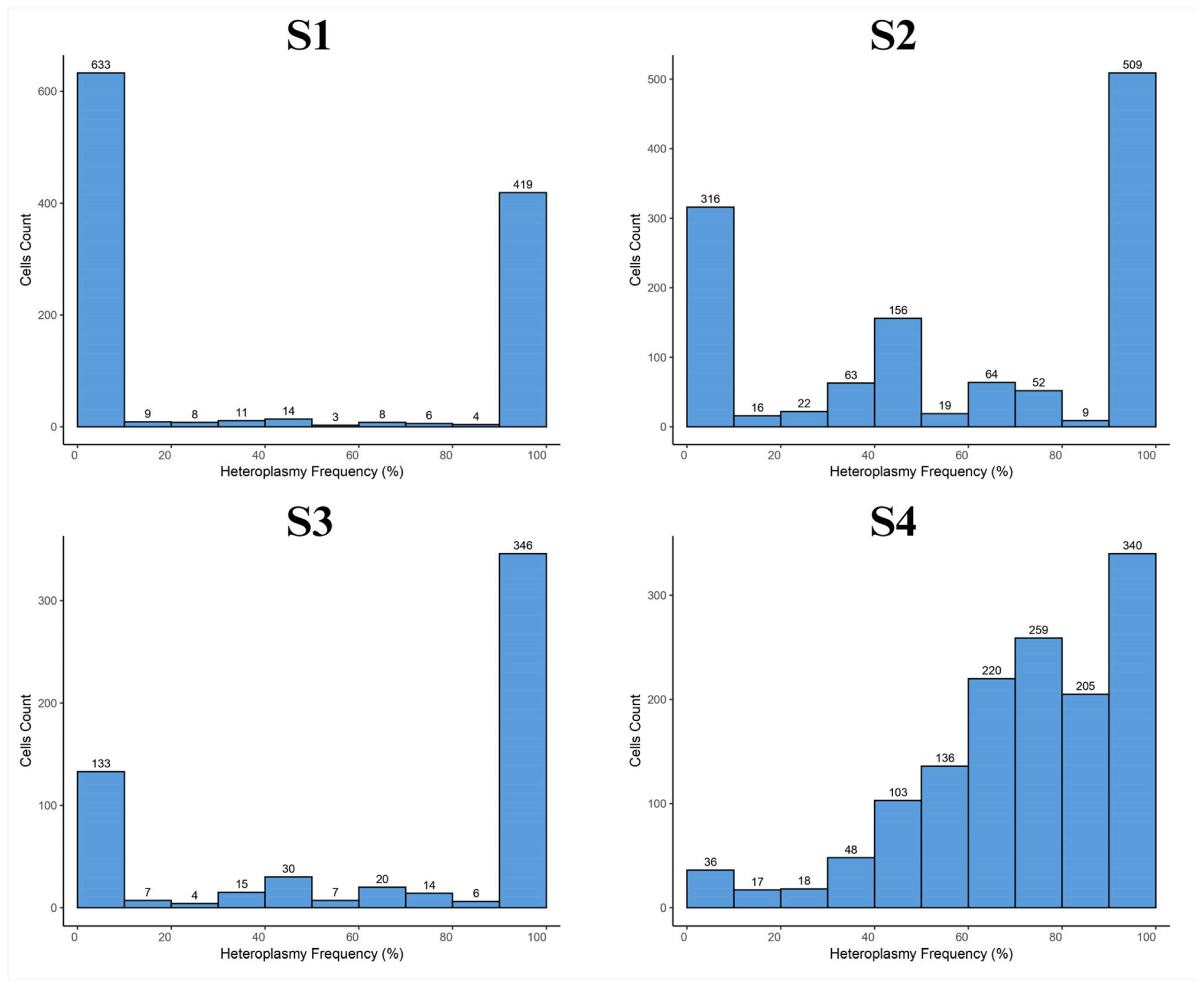
**Distribution of per-cell heteroplasmy across four patient fibroblast samples.** The graphs showed the distribution of per-cell heteroplasmy in the samples S1–S4. The *y*-axis represents the number of cells, and the *x*-axis shows heteroplasmy frequency in percentage with 10% histogram bins.

Despite the tested samples had a mean RNA heteroplasmy between 38% and 69%, analysis of per-cell heteroplasmy distribution revealed that S1–S3 samples showed a bimodal distribution with most cells near homoplasmic wild-type (heteroplasmy 0–10%) or homoplasmic mutant status (heteroplasmy 90–100%) and few cells displaying intermediate percentages of mutation load (Figure 2), while S4 showed a more “Gaussian” distribution plus a peak for near homoplasmic mutant status. The same patterns were observed when the analysis was restricted to cells with higher coverage (5× or 10×; Figure S1), indicating that the results were not driven by low-coverage artifacts.

To assess whether the distinctive distribution observed in S4 reflected altered transcript abundance, we directly compared per-cell abundance of *MT-ND4* and *MT-ND5* between high-mutant heteroplasmy (90–100%) and low-mutant heteroplasmy (0–10%) populations across all four samples. Neither raw read counts nor log–normalized expression values for *MT-ND4* or *MT-ND5* differed significantly between the two groups in any patient line (all FDR-adjusted *p*-values > 0.4; Tables S2 and S3), including S4 (Figure S2).

### 3.4. Transcriptomics Effects of Different RNA Heteroplasmy Levels in the Same Subject/Genetic Background

**Figure 3:**
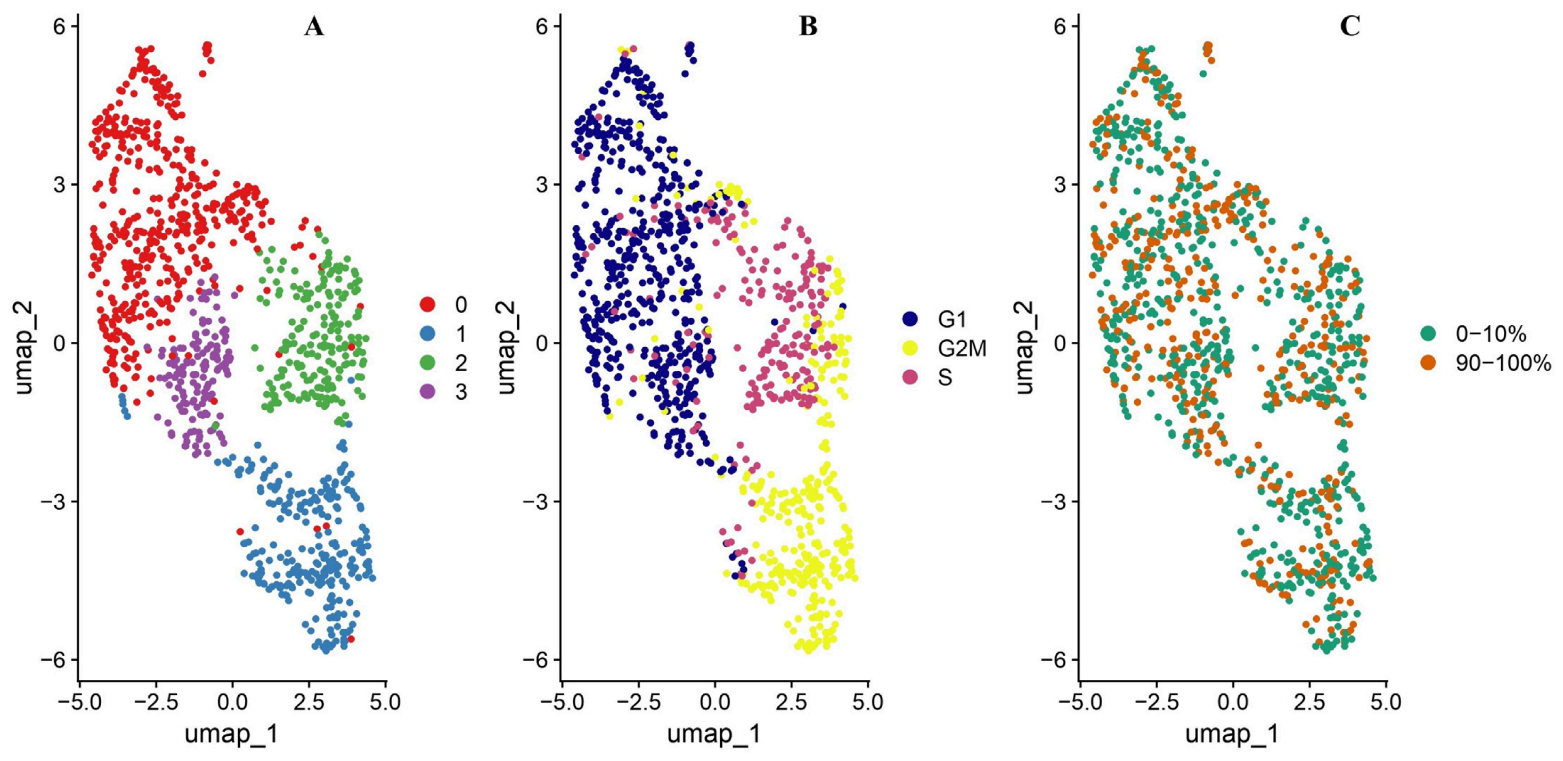
**UMAP embedding of sample S1 single-cell transcriptomes.** (**A**) UMAP clustering with resolution = 0.3. (**B**) Cluster composition using Seurat’s CellCycleScoring: each cell was assigned a cell-cycle phase (G1, S, G2/M). (**C**) Cells with high (90–100%, in orange) and low (0–10%, in teal) RNA heteroplasmy are broadly intermingled across the embedding, indicating that heteroplasmy level does not drive the main transcriptional variation.

To test whether high vs. low heteroplasmy defines discrete transcriptional states, we independently analyzed each cell line using UMAP clustering, a data visualization technique that groups cells with similar gene expression profiles into distinct clusters. Here, we show sample S1 as the most representative example. Using a resolution of 0.3, all cells were stratified into four UMAP embedding clusters. (Figure 3A). To reduce confounding from cell-cycle–driven transcriptional variation and improve the biological interpretability of our clustering [18], we assigned each cell to a cell-cycle phase and used phase composition, together with marker and enrichment analyses, to annotate clusters. These four UMAP clusters therefore reflect distinct cell-cycle states rather than stable cell-type differences (Figure 3B). CellCycleScoring assigned clusters 0 and 3 predominantly to G1 (~81% and ~91%), cluster 1 to G2/M (~87.5%) and cluster 2 to S (~69.7%). Accordingly, cluster-specific top markers (e.g., NEK2, CDC20, DLGAP5, AURKA) and preranked GSEA show strong G2/M enrichment for cluster 1 (G2/M NES ≈ +2.87, padj ≪ 0.001) and S-phase enrichment for cluster 2 (S NES ≈ +2.09, padj ≪ 0.001), while clusters 0 and 3 are depleted of cell-cycle genes (large negative NES), consistent with G1/quiescent states. Low- and High-heteroplasmy cells (Figure 3C) remained fully interspersed across principal clusters, demonstrating that heteroplasmy level is not the defining factor in global transcriptomic clustering. Although the sharpness of cluster boundaries varied with cell number, a consistent correspondence between clusters and cell-cycle phases was maintained, and the same pattern of intermixing between high- and low-heteroplasmy cells was also observed in the other samples (Figure S3).

**Figure 4:**
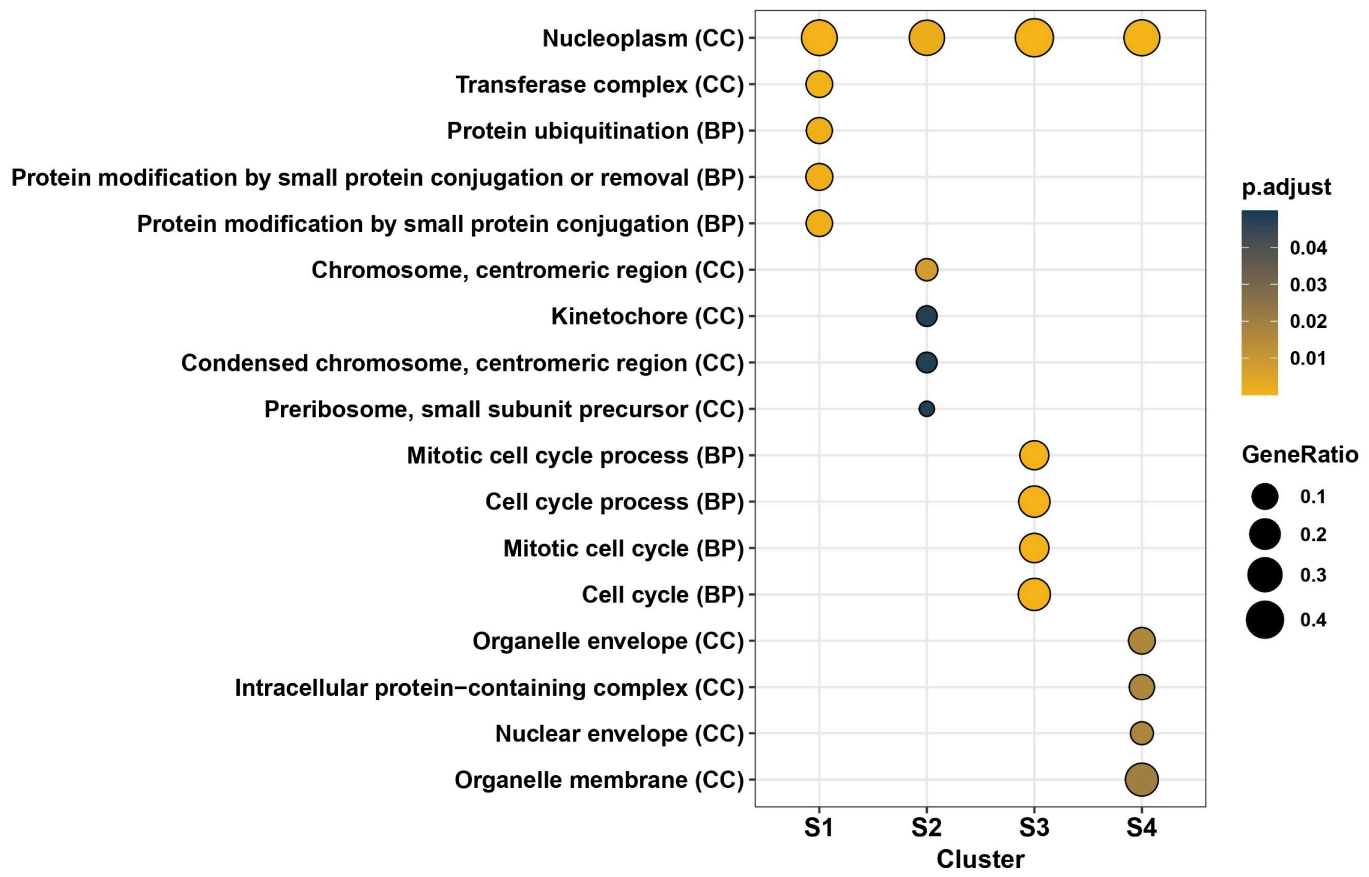
**Comparative GO enrichment dot plot across four fibroblast samples (S1–S4) stratified by extreme heteroplasmy (>90% vs. 0–10%).** Each point represents a GO term significantly enriched (adjusted *p*-value < 0.05, minGSSize = 5) in the DEGs of the corresponding sample. The *y*-axis lists the top 5 GO categories ranked by adjusted *p*-value, and the *x*-axis indicates aggregated samples. Point size reflects the number of DEGs contributing to the term, and color gradient represents the magnitude of *p*-adjust values.

To assess whether each cell line shows a distinct transcriptional response to high heteroplasmy (>90%) at the individual-sample level, we performed sample-wise differential expression analysis and pathway enrichment analysis (Figure 4).

Only genes expressed in at least 20% of cells were retained. Because no protein-coding gene survived a transcriptome-wide FDR correction, we defined candidate DEGs as those with a nominal LRT *p*-value (*p*(>Chisq) < 0.05) from the hurdle model. To further characterize modes of regulation, we extracted logFC, discrete (β_D), and continuous (β_C) hurdle model coefficients. For gene ontology (GO) pathway enrichment, we considered only terms with adjusted *p*-value < 0.05 and 5 minimal gene sizes annotated by the ontology term for testing.

High-heteroplasmy cells from the S1 sample showed enrichment in ubiquitin-related and protein quality control functions. By contrast, the S2 sample showed enrichment almost exclusively in chromosome structural components, suggesting a potential delay or dysregulation of the chromosomal segregation machinery under high heteroplasmy, which may impair faithful cell division. S3 cells displayed strong enrichment for mitotic and cell-cycle processes, indicating a robust involvement of cell-division programs and checkpoint regulators. Finally, S4 displays yet another set of enriched categories: nuclear and organelle envelope components and intracellular protein complexes. Although nucleoplasm is a top-enriched cellular component in every sample, this reflects different nuclear programs being engaged in each case rather than a single conserved nuclear response. Indeed, no single gene is shared by all four sets. Reducing the minimal gene size to 2 for testing reveals additional GO enrichments in the four samples (Figure S4). All four lines each exhibit a distinct transcriptional fingerprint under high heteroplasmy. These sample-specific responses underscore the heterogeneous cellular strategies that may be adopted to cope with mitochondrial genome imbalance. Nevertheless, inter-individual genetic variability on the nuclear background, as well as intrinsic differences in cell culture status or conditions, may influence the transcriptional profiles observed across the tested samples.

To specifically assess the impact of extremely high heteroplasmy on the expression of genes with mitochondrial localization/function, we next performed a targeted enrichment of our nominal DEGs against the MitoCarta3.0 gene set, aiming to identify candidate-driven mitochondrial gene signatures characterizing each mutant background (Table S4).

In S1, we observed coordinated upregulation of genes involved in Complex IV structure or assembly (*COX7A1*, *SCO1*) and mitochondrial translation (*MRPS27*, *MTG2*), together with an increased proportion of cells expressing *COX10*. By contrast, *SLC25A20* and *LIAS* were downregulated both in expression frequency and magnitude. In S2, a set of mitochondrial DNA maintenance and translation factors (e.g., *POLRMT*, *LIG3*, *MRPL30*, *DNAJA3*) and metabolic enzymes (e.g., *ACAD10*, *IDH3A*) genes showed changes almost entirely driven by the fraction of cells expressing each gene rather than by per-cell transcript abundance. Notably, the Complex I subunit gene *MT-ND4L* expression was detected in fewer cells under high heteroplasmy, whereas the Complex III assembly factor *UQCC1* gene exhibited reduced transcript abundance per cell.

In S3, we observed combined up-regulation in both the fraction of expressing cells and per-cell abundance—of the ATP synthase subunit *MT-ATP8* (Complex V) and proteostasis/import genes (*CLPP*, *ATAD3A*, *TOMM40*), along with down-regulation of the replication factor *POLG* and antioxidant enzyme *OXR1* in either expression frequency or magnitude. Finally, the S4 sample reveals a highly nuanced mitochondrial rewiring rather than a uniform shutdown. At the level of Complex I, we see a concerted down-regulation of core subunit genes—*NDUFS1* and its assembly factor *NDUFAF7* both lose expression in more cells and at lower levels—while *NUBPL* deviates from the trend with a subtle, continuous-only increase in transcript output among its remaining expressers. Complex II follows suit: both *SDHA* and *SDHAF1* genes decline in frequency and magnitude, pointing to diminished succinate dehydrogenase activity. Downstream, we detect no classical subunit hits for Complex III, IV, or V.

Subsequent interrogation of our nominal DEGs against the MitoCarta3.0 MitoPathways repertoire revealed that, although all four fibroblast lines differentially express mitochondrial genes under extreme (>90%) heteroplasmy, they do so with largely distinct repertoires. S2 shows the highest mitochondrial burden (8.2% of its 256 DEGs), closely followed by S4 (7.9% of its 391 DEGs), and then S1 and S3 at roughly 6% each of 332 and 533 DEGs, respectively. When we examine the overlap of these MitoCarta hits, the vast majority are unique to a single sample. Only a handful of genes are shared between pairs of lines, while no mitochondrial gene is differentially expressed in more than three samples.

**Figure 5:**
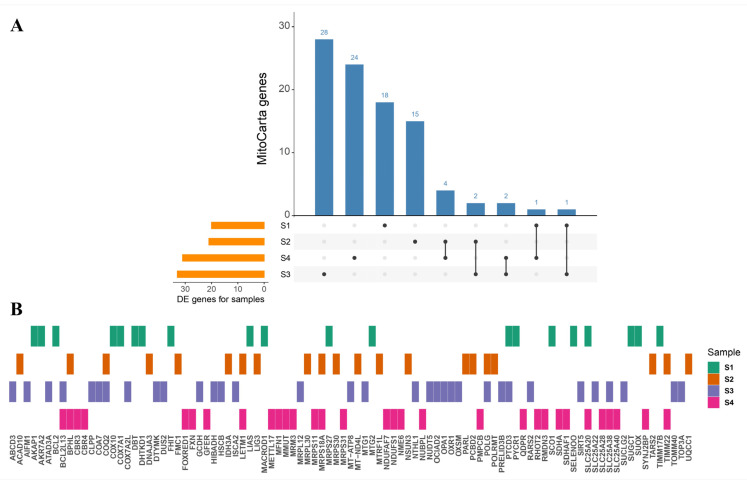
**Intersection and presence/absence of MitoCarta3.0–annotated DEGs across four fibroblast samples under extreme heteroplasmy.** (**A**) The horizontal light orange bars indicate the total number of mitochondrial DEGs per sample, and the vertical blue bars show the sizes of each intersection set. Dots and connecting lines beneath indicate which sample combinations contribute to each intersection. (**B**) Each vertical tile represents a single MitoCarta DEG, colored by the samples in which it is differentially expressed. Tiles are grouped by sample membership, enabling visual identification of shared versus unique mitochondrial gene signatures. Colors: green = S1, orange = S2, purple = S3, magenta = S4.

A complementary presence/absence map groups each gene by sample membership, clustering translation factors, OXPHOS subunits, and quality-control components, underscoring the sample-specific nature of the mitochondrial response to extreme heteroplasmy (Figure 5).

### 3.5. Transcriptomics Effects of High Heteroplasmy for Complex I mtDNA

Having characterized these sample-specific signatures, we next aggregated cells from all four samples with complex I subunit variants to uncover more subtle transcriptional effects of extreme heteroplasmy. We divided all cells from the 4 subjects S1–4 into high vs. low heteroplasmy cell groups and performed single-cell differential expression analysis using the MAST hurdle model, with plate-level pooling and sample identity included as a fixed covariate.

In our analysis, we detected 502 genes whose expression differs between high- and low-heteroplasmy cells (LRT *p* < 0.05). Of these, only about 9% (42/502) are annotated in MitoCarta3.0 (Table S5). This proportion is comparable to, or slightly higher than, the per-sample enrichments we observed previously (6–8%), indicating that pooling cells does not reveal a stronger core mitochondrial response, but rather recovers a modest “common” set of mitochondrial genes.

The mitochondrial hits encompass a mix of up- and down-regulation across OXPHOS complexes and their supporting machinery. In Complex I, for instance, some core subunit genes—like *NDUFA9*—showed increased transcript levels, while other key components—like subunits *MT-ND5* and *NDUFV3* and assembly factor *NDUFAF7* genes—are both expressed in fewer cells and at lower levels. A similar pattern emerges in Complex III: the assembly-factor gene *UQCC1* is shut off in a subset of cells, even as other Complex III subunits remain unaffected, pointing to targeted remodeling of the bc1 machinery rather than global disassembly. Conversely, complex IV shows a differentiated strategy. The oxidase subunits *SCO1* and *SCO2* are broadly up-regulated, suggesting an effort to shore up electron-transfer capacity. In contrast, cytochrome c (*CYCS*) is silenced in many cells, and only a fraction retains expression of *COX7A1*, a key assembly scaffold, suggesting that Complex IV assembly is preserved in some cells, while compromised or absent in others. Finally, Complex V shows a modest, continuous-only drop in *MT-ATP8* transcript levels in expressing cells, suggesting nuanced throttling of ATP synthase output rather than a blanket shutdown.

Outside the core OXPHOS modules, expression of proteins related to mitochondrial translation is also rebalanced. For instance, *MRPL55* and *MRPL57* rise in cells that keep them on, indicating selective up-regulation of mitoribosome biogenesis. Meanwhile, *CHCHD3* and *MICOS10*, encoding components of the inner-membrane organizing system, are pulled back, suggesting a contraction of membrane architecture support in some cells (Table S5).

The integration of data across complex I-deficient patients suggests the emergence of two distinct profiles: cells that sustain mitochondrial gene expression fine-tune specific OXPHOS subunits and translation machinery, whereas others quietly abandon key assembly factors. This heterogeneous rewiring underscores the complexity of cellular adaptation to extreme heteroplasmy.

**Figure 6:**
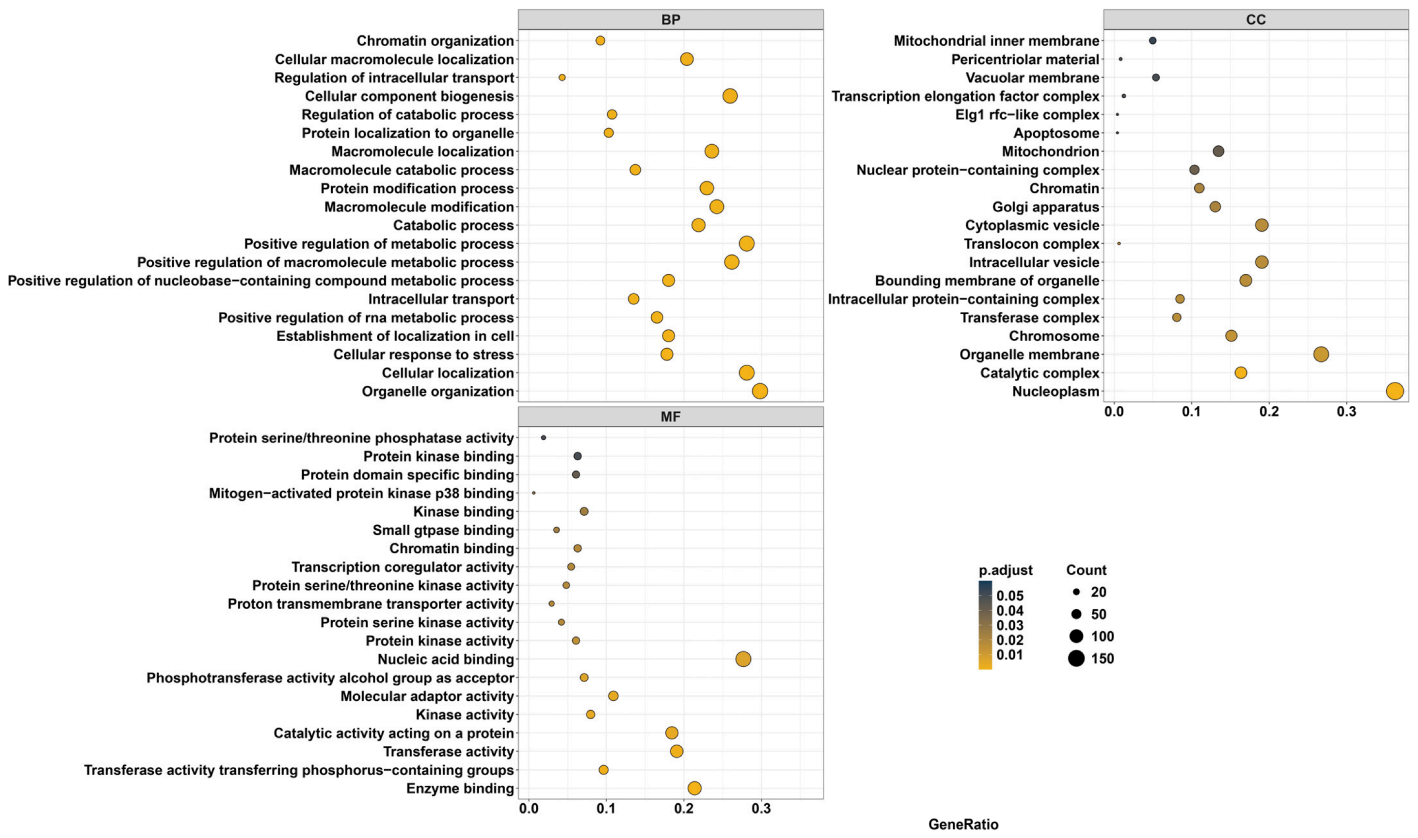
**Ontology-stratified GO enrichment summary for 502 genes differentially expressed between high- and low-heteroplasmy cells across four fibroblast lines**. Dot plot of GO enrichment results (Benjamini–Hochberg adjusted *p* < 0.05, minGSSize = 2) split by ontology: Biological Process (BP), Cellular Component (CC), and Molecular Function (MF). The top 20 terms per ontology (ranked by adjusted *p*-value are shown. The dot position on the *x*-axis represents the GeneRatio, the dot size corresponds to the number of genes contributing to each term (Count), and color encodes the adjusted *p*-value.

Functional enrichments of total DEGs across all GO domains, and KEGG pathways—each tested by Fisher’s exact test with Benjamini-Hochberg adjustment at *p* < 0.05 and two minimal sizes of genes annotated by the Ontology term for testing—highlight a widespread reprogramming across organelle organization and subcellular localization, metabolic processes, nucleic acid binding (Figure 6).

At the cellular-component level, nuclear and broader organelle/membrane categories feature prominently, suggesting targeted remodeling of organelle architecture. In addition to these findings, when we turn to the KEGG database, the only significant hit is oxidative phosphorylation. Together, these shared enrichments highlight that, beyond transport and proteostasis, fine-tuning of energy-production machinery itself is a central shared feature of the high-heteroplasmy state, their interpretation, as well as the experimental conclusions that can be drawn.

## 4. Discussion

Using 10x Chromium 3′ scRNA-seq data, we demonstrated that mitochondrial RNA heteroplasmy can be estimated at single-cell resolution and that extreme heteroplasmy (>90%) is associated with both sample-specific transcriptional signatures and shared stress-response axes. scRNA heteroplasmy estimates generally agreed with bulk RNA and DNA measurements, while per-cell analyses revealed marked heterogeneity within cell lines, including bimodal heteroplasmy distributions in three of four samples. Sample-wise and pooled transcriptomics analyses identified distinct mitochondrial and non-mitochondrial pathways involved in the response to high heteroplasmy.

As reported, our single-cell coverage analyses of mtDNA-encoded transcripts showed a strong 3′-end bias inherent to the 10x Chromium 3′ scRNA-seq chemistry, which limits the ability to uniformly capture long or poorly polyadenylated mitochondrial transcripts. Owing to the limited amount of starting material in single cells, protocols are optimized for efficiency rather than completeness. This leads to shallow coverage and a focus on quantifying gene expression rather than reconstructing full-length transcripts. Most scRNA-seq protocols (e.g., 10x Chromium 3′ kit) are designed to capture polyadenylated mRNA using oligo(dT) primers. This means that reverse transcription starts at the poly(A) tail, which is located at the 3′-end of the transcript. As a result, only the 3′-end fragments of the transcripts were reverse-transcribed and sequenced. In particular, *MT-ND5*—the largest mtDNA-encoded gene at ~1.8 kb—shows the lowest and most uneven coverage, likely reflecting both its length and potential secondary structure hurdles to reverse transcriptase processivity, leading to drop-off and incomplete cDNA synthesis. Likewise, variable poly(A) tail length or stability of mitochondrial mRNA may further reduce capture efficiency. Longer transcripts like *MT-ND5* may not be fully captured, particularly if the poly(A) tail is distant from the coding region. Furthermore, mitochondrial transcripts, including *MT-ND5*, may undergo variable or inefficient polyadenylation, affecting their capture by oligo(dT) primers. These technical constraints make the reliability of per-cell heteroplasmy estimates dependent on the variant position within the transcript and its local sequence context. Previous studies based on single-cell ATACseq have shown that neither sequencing depth nor coverage at the variant position influences measurement of heteroplasmy [19]. However, it should be noted that low coverage may cause overestimation of the extreme heteroplasmy values (0% and 100%). Despite these potential biases, the per-cell read counts obtained for *MT-ND4* and *MT-ND5* were sufficient to stratify cells into “low” (0–10%) and “high” (90–100%) heteroplasmy classes.

Another limitation to consider for the tested approach is that non-polyadenylated mitochondrial RNAs, including tRNAs and rRNAs, are not captured in this workflow and would require different methodological strategies. The scRNAseq coverage profile showed few reads located in the D-loop region. Adapter–dimer artifacts or mispriming during library preparation can create chimeric reads that may map to the D-loop. Additionally, 3′ library preparation kits can capture non-polyA fragments at low levels. mtDNA is transcribed as long polycistronic units that extend into the D-loop region; furthermore, D-loop gives rise to several mtDNA-encoded non-coding RNAs, including long ncRNAs [20]. It is thus difficult to distinguish between the D-loop low coverage due to real non-coding mtRNAs and non-specific (adapter dimers) reads, which may align to repetitive mitochondrial regions, inflating apparent D-loop coverage.

Despite discrepancies that may occur between mtDNA heteroplasmy and allele fractions inferred from transcript reads because variant-harbouring mitochondrial RNAs can be differentially unstable, degraded, or selectively transcribed/processed, our findings indicate that there are no active mechanisms against mutant mtDNA transcripts, at least for the investigated variants. Additionally, the concordance between DNA and RNA heteroplasmy estimates suggests that mitochondrial C→T RNA editing, which was not considered in our analysis, is unlikely to significantly affect heteroplasmy measurements. The per-cell bimodal heteroplasmy distributions observed in S1–S3—cells concentrated near 0–10% or 90–100%—may reflect intracellular segregation dynamics of mtDNA, selection for or against particular mitochondrial genotypes in culture, or differential turnover of mitochondrial genomes across subpopulations. The absence of significant differences in *MT-ND4*/*MT-ND5* transcript abundance between the High and Low groups argues against a simple model in which mutant transcripts are universally (de)stabilized or over/under-transcribed across samples. Notably, a similar bimodal distribution was found by single-cell DNA analysis (ATAC-seq) in fibroblasts from a subject harboring the m.3243A>G variant; conversely, five other cell lines with the same genetic defect showed a Gaussian distribution or a highly skewed distribution towards nearly homoplasmic values [21].

Our single-sample analysis revealed remarkable diversity in mitochondrial responses to extreme heteroplasmy. Some lines (S1, S3) mount a “downstream compensation” by up-regulating Complex IV subunits and mitochondrial translation factors, presumably to boost electron flux around a deficient Complex I. Others (S2) appear to shift resources toward genome maintenance at the expense of respiratory-chain structure, as evidenced by selective suppression of Complex I/III components and upregulation of mtDNA maintenance factors. This pattern suggests a strategically reduced priority of respiratory assembly in favor of preserving mitochondrial genome integrity. In the S4 line, we observed targeted silencing of OXPHOS genes across multiple complexes. These contrasting strategies—from targeted downstream boosting (S1, S3), through genome-focused resource allocation (S2), to uniform shutdown and limited compensation (S4)—highlight how fibroblasts deploy sample-specific OXPHOS programs to contend with the high burden of Complex I variants.

When we aggregated all four sample lines into a single plate-level pooling model, a coherent cross-cutting stress response emerged. GO Biological Process enrichments center on chromatin reorganization and processes linked to subcellular localization and transport, indicating that high heteroplasmy universally triggers tighter cell-cycle surveillance. Simultaneously, RNA-processing pathways are upregulated, suggesting enhanced post-transcriptional quality control. The terms related to mitochondria, vacuolar membranes, and intracellular vesicles suggest an activation of organellar turnover and vesicular traffic: this is consistent with intensified processes of disposal/recycling of damaged organelles. KEGG hits about oxidative phosphorylation likely reflect perturbation of mitochondrial and membrane-associated gene sets. Together, these shared axes–cell-cycle regulation, RNA quality control, selective organelle turnover, and genome-integrity enforcement–provide a framework for understanding possible common features of the high-heteroplasmy state, even as individual lines diverge in their mitochondrial-specific adaptations. However, dedicated functional validation studies are required to experimentally support these findings. The aim of this manuscript, however, is to demonstrate that scRNA-seq can serve as a rapid approach to obtain an initial overview of transcriptional alterations associated with specific mtDNA variants, and experimental validation lies beyond the scope of the present work. Such validation would necessitate the generation of cell lines exhibiting extreme heteroplasmy levels (e.g., transmitochondrial cybrids), which is labor-intensive and time-consuming. In addition, cybrid models, for instance, do not capture the potential contribution of the patient’s nuclear genetic background.

Building on these insights, we demonstrate a novel “virtual homoplasmic clone” approach: by stratifying cells from the same line into low- and high-heteroplasmy groups, we effectively create in silico cohorts of homoplasmic wild-type or mutant cells with the same genetic background that can be interrogated at scale. This strategy not only potentially enables robust differential expression analyses within individual patient lines but also provides a high-throughput framework for prioritizing mtDNA variants of uncertain significance for downstream validation and probing genotype–phenotype relationships within a controlled, in-silico framework. Extending this workflow to larger cohorts of patient specimens (and possibly to research models such as organoids) and integrating it with multimodal single-cell data will help the dissection of pathogenic versus benign mitochondrial variants and illuminate the cellular strategies that buffer or amplify mitochondrial dysfunction.

## 5. Conclusions

Our study shows that single-cell resolution of mtDNA heteroplasmy, coupled with a reproducible computational workflow, offers a powerful new window into cellular mitochondrial variability. At the aggregate level, RNA-derived heteroplasmy estimates agree with those obtained from DNA and bulk RNA; however, cell-by-cell analysis reveals a more nuanced reality: most cells tend to be at the extremes (close to wild-type or mutant homoplasmy), whereas only a small fraction shows intermediate mutational loads. This pattern highlights how the same average heteroplasmy value can conceal very different distributions at the cellular level, with important implications for understanding the penetrance and phenotypic variability of mitochondrial diseases. Although high heteroplasmy is associated with transcriptional remodeling, these responses are largely sample-specific, with each fibroblast line displaying a distinct program of mitochondrial adaptation. Such diversity implies that the functional impact of a given mtDNA variant is likely strongly modulated by nuclear background and cellular context rather than being governed by a single, uniform mechanism.

## Figures and Tables

**Table 1:** Comparison of heteroplasmy levels in genomic DNA and single-cell RNA (scRNA) for four mitochondrial DNA variants.

Sample	Gene	Variant	DNA Het.	Bulk RNA Het.	scRNA Het. *	ScRNA Mean Cov.	n Cells **	Alt Reads ***	Total Reads ***
S1	*MT-ND5*	m.13063G>A	38%	36%	38%	5.61	1115	2391	6261
S2	*MT-ND5*	m.13514A>G	60%	64%	58%	3.11	1226	2208	3821
S3	*MT-ND5*	m.13513G>A	63%	65%	69%	3.11	582	1247	1814
S4	*MT-ND4*	m.11777C>A	68%	68%	72%	34.90	1382	32,960	45,396

Het.: heteroplasmy; Cov.: coverage. * Heteroplasmy percentages for scRNA were calculated as the ratio of pooled alternate-allele reads to pooled total read depth across all cell-associated barcodes with coverage ≥2 reads at the variant position (pseudobulk estimate). ** n Cells: total number of cells (contributing barcodes). *** Alt Reads and Total Reads: pooled read counts for the alternative allele or both alternative and wt alleles in the specific variant positions.

## Data Availability

Raw data are available at the repository Zenodo: 10.5281/zenodo.18197171. Further data related to this study are available on request from the corresponding author due to privacy/consent reasons.

## Supplementary Materials

The following supporting information can be downloaded at: https://www.mdpi.com/article/10.3390/cells15151403/s1, They include: Figure S1: Distribution of per-cell heteroplasmy across four patient fibroblast samples, considering cells with ≥5× or 10 × reads in the sample-specific mtDNA variant position; Figure S2: Raw and log-normalized expression of mitochondrial genes *MT-ND4* and *MT-ND5* stratified by heteroplasmy category (0–10% vs. 90–100%) across four patient fibroblast samples (S1–S4); Figure S3: Per-sample UMAP embeddings for fibroblast lines S2–S4 (one row per sample); Figure S4: Ontology-stratified GO enrichment across four fibroblast lines (S1–S4) under extreme mitochondrial heteroplasmy (>90% vs. 0–10%). Table S1: Summary of single-cell RNA sequencing quality metrics for the four samples; Table S2: Key statistics (Wilcoxon test, FDR-adjusted *p* > 0.4 for all comparisons) in raw counts; Table S3: Key statistics (Wilcoxon test, FDR-adjusted *p* > 0.4 for all comparisons) in normalized data counts; Table S4: MitoCarta3.0-annotated differentially expressed genes in each sample under extreme heteroplasmy (>90% vs. 0–10%); Table S5: MitoCarta3.0-annotated differentially expressed genes in pooled samples S1–S4 between high- and low-heteroplasmy (>90% vs. 0–10%, LRT *p* < 0.05).

## Abbreviations

The following abbreviations are used in this manuscript:

DEG	Differentially expressed gene
GO	gene ontology
mtDNA	mitochondrial DNA
PCA	Principal Component Analysis
scRNA-seq	single-cell RNA sequencing
UMAP	Uniform Manifold Approximation and Projection
