# Supplementary material for: Exploring Heteroplasmic Variants in mtDNA: Insights from Single-Cell Transcriptomics
Source: Cells. 2026 Aug 3;15(15):1403. doi: 10.3390/cells15151403 (PMC13464780; doi:10.3390/cells15151403)
Supplement: Supplementary file 1 [file cells-15-01403-s001.zip › cells-4424061-supplementary.pdf]

Exploring Heteroplasmic Variants in mtDNA: Insights from Single-Cell Transcriptomics

Marco Barresi [1], Ivano Di Meo [1], Alessia Nasca [1], Eleonora Lamantea [1], Andrea Legati [1] and Daniele Ghezzi [1,2,*]

[1] Unit of Medical Genetics and Neurogenetics, Fondazione IRCCS Istituto Neurologico Carlo Besta, 20126 Milan, Italy; marco.barresi@istituto-besta.it (M.B.); ivano.dimeo@istituto-besta.it (I.D.M.); alessia.nasca@istituto-besta.it (A.N.); eleonora.lamantea@istituto-besta.it (E.L.); andrea.legati@istituto-besta.it (A.L.)

[2] Department of Pathophysiology and Transplantation (DEPT), University of Milan, 20122 Milan, Italy

* Correspondence: daniele.ghezzi@istituto-besta.it or daniele.ghezzi@unimi.it

Supplementary Materials

Supplementary material includes 5 tables and 4 figures.

Supplementary Table S1. Summary of single-cell RNA sequencing quality metrics for the four samples

Metric*	S1	S2	S3	S4
Estimated Number of Cells	2,614	5,120	2,826	1,919
Number of Reads	422,297,256	467,712,174	506,417,189	436,004,451
Mean Reads per Cell	161,552	91,350	179,199	227,204
Median Genes per Cell	5,950	5,364	6,304	7,125
Total Genes Detected	16,653	16,863	16,914	16,943

**Metrics were generated using Cell Ranger (10x Genomics).*

Supplementary Table S2. Key statistics (Wilcoxon test, FDR-adjusted p > 0.4 for all comparisons) in raw counts

sample	gene	med_90_100	med_0_10	log_2FC[1]	p_value	p_adj
S1	*MT-ND4*	136	138	-0.02106	0.151703	0.466904
S1	*MT-ND5*	22	23	-0.06413	0.117216	0.466904
S2	*MT-ND4*	145.5	147.5	-0.0197	0.625216	0.725321
S2	*MT-ND5*	27	28	-0.05247	0.422222	0.675556
S3	*MT-ND4*	137.5	135	0.026472	0.370624	0.675556
S3	*MT-ND5*	12	13	-0.11548	0.175089	0.466904
S4	*MT-ND4*	169	174.5	-0.0462	0.854571	0.854571
S4	*MT-ND5*	22	24.5	-0.15528	0.634656	0.725321

[1] log_2FC calculated as $log_2(median_{90-100}\ \% / median_{0-10}\ \%)$.

Supplementary Table S3. Key statistics (Wilcoxon test, FDR-adjusted p > 0.4 for all comparisons) in normalized data counts.

sample	gene	med_90_100	med_0_10	log_2FC[1]	p_value	p_adj
S1	*MT-ND4*	3.53534	3.510103	1.951595	0.533223	0.73173
S1	*MT-ND5*	1.830181	1.867826	2.030465	0.172276	0.73173
S2	*MT-ND4*	3.77063	3.768152	1.970173	0.593752	0.73173
S2	*MT-ND5*	2.151444	2.198687	1.892807	0.317676	0.73173
S3	*MT-ND4*	3.437477	3.423126	1.936264	0.927116	0.927116
S3	*MT-ND5*	1.284314	1.35311	2.94379	0.210773	0.73173
S4	*MT-ND4*	3.306982	3.203331	1.968943	0.49022	0.73173
S4	*MT-ND5*	1.539263	1.530917	2.505327	0.640264	0.73173

[1] log_2FC calculated as $log_2(median_{90-100}\ \% / median_{0-10}\ \%)$.

Supplementary Table S4. MitoCarta3.0–annotated differentially expressed genes in each sample under extreme heteroplasmy (> 90 % vs. 0–10 %).

S1 sample										
**primerid**	**Pr(>Chisq)**	**coef**	**ci.hi**	**ci.lo**	**fdr**	**fold_change[1]**	**log_2FC[2]**	**β_D**	**β_C**	**category**
AKAP1	0.0297	0.1036	0.1810	0.0261	0.9978	1.1091	0.1494	0.3393	0.0329	up
AKR7A2	0.0100	0.2068	0.3422	0.0714	0.9978	1.2297	0.2983	0.3814	0.1497	up
BCL2	0.0238	0.0072	0.1021	-0.0877	0.9978	1.0072	0.0104	0.1798	-0.1555	D_driver
COX10	0.0015	0.1008	0.1794	0.0223	0.9978	1.1061	0.1455	0.4258	-0.0548	D_driver
COX7A1	0.0042	0.1960	0.3348	0.0572	0.9978	1.2165	0.2828	0.4398	0.0063	up
DBT	0.0319	-0.0194	0.0500	-0.0888	0.9978	0.9808	-0.0280	-0.1788	0.0810	D_driver
DHTKD1	0.0373	0.0570	0.1348	-0.0208	0.9978	1.0587	0.0822	0.2748	-0.0558	D_driver
FHIT	0.0121	-0.0543	0.0524	-0.1611	0.9978	0.9471	-0.0784	0.0733	-0.1799	C_driver
LIAS	0.0413	-0.0831	-0.0151	-0.1511	0.9978	0.9203	-0.1199	-0.3545	0.0045	D_driver
MACROD1	0.0403	0.0434	0.1470	-0.0602	0.9978	1.0443	0.0626	-0.0704	0.1019	C_driver
MRPS27	0.0281	0.0582	0.1613	-0.0450	0.9978	1.0599	0.0839	0.3075	-0.0607	D_driver
MTG2	0.0098	0.1124	0.2011	0.0236	0.9978	1.1189	0.1621	0.2030	0.0932	up
PTCD3	0.0050	0.0049	0.1042	-0.0944	0.9978	1.0049	0.0071	-0.2264	0.1086	C_driver
PYCR1	0.0496	-0.0095	0.0716	-0.0907	0.9978	0.9905	-0.0138	0.0936	-0.1004	C_driver
SCO1	0.0207	0.1395	0.2383	0.0406	0.9978	1.1496	0.2012	0.3453	0.0424	up
SELENOO	0.0232	-0.0219	0.0683	-0.1120	0.9978	0.9783	-0.0316	0.1020	-0.0934	C_driver
SLC25A20	0.0091	-0.0932	-0.0259	-0.1605	0.9978	0.9110	-0.1345	-0.2914	-0.0727	down
SUGCT	0.0384	-0.1397	-0.0287	-0.2507	0.9978	0.8696	-0.2015	-0.2163	-0.1130	down
SUOX	0.0377	0.0755	0.1355	0.0155	0.9978	1.0784	0.1090	0.3920	0.0065	up
TIMM17B	0.0204	-0.1076	0.0053	-0.2204	0.9978	0.8980	-0.1552	-0.3776	0.0297	D_driver

S2 sample										
**primerid**	**Pr(>Chisq)**	**coef**	**ci.hi**	**ci.lo**	**fdr**	**fold_change[1]**	**log2FC[2]**	**β_D**	**β_C**	**category**
ACAD10	0.0455	0.0403	0.1045	-0.0239	0.9999	1.0411	0.0581	0.3012	-0.0683	D_driver
BPHL	0.0097	0.0824	0.1626	0.0022	0.9999	1.0859	0.1189	0.4132	-0.0675	D_driver
COQ2	0.0087	-0.0843	0.0244	-0.1931	0.9999	0.9191	-0.1217	-0.3746	0.0809	D_driver
DNAJA3	0.0066	0.0398	0.1283	-0.0487	0.9999	1.0406	0.0574	0.2836	-0.1141	D_driver
FMC1	0.0109	-0.0370	0.0469	-0.1209	0.9999	0.9637	-0.0533	-0.2613	0.0935	D_driver
IDH3A	0.0284	-0.0389	0.0599	-0.1378	0.9999	0.9618	-0.0562	-0.2557	0.0916	D_driver
LETM1	0.0446	-0.0028	0.0903	-0.0959	0.9999	0.9972	-0.0040	-0.1425	0.0991	D_driver
LIG3	0.0115	0.0932	0.1667	0.0197	0.9999	1.0977	0.1344	0.4614	-0.0388	D_driver
MRPL30	0.0248	0.0277	0.1141	-0.0588	0.9999	1.0281	0.0399	0.2250	-0.0991	D_driver
MRPS18A	0.0118	-0.1383	-0.0197	-0.2570	0.9999	0.8708	-0.1996	-0.4367	0.0236	D_driver
MRPS30	0.0466	-0.0377	0.0632	-0.1385	0.9999	0.9630	-0.0543	-0.2379	0.0797	D_driver
MT-ND4L	0.0330	-0.0149	0.0984	-0.1282	0.9999	0.9852	-0.0216	-1.7373	0.0367	D_driver
MTRF1L	0.0058	0.1114	0.2112	0.0117	0.9999	1.1179	0.1608	0.1387	0.1358	up
NSUN3	0.0192	-0.0556	0.0178	-0.1290	0.9999	0.9459	-0.0802	-0.1355	-0.1123	down
PARL	0.0453	-0.0522	0.0432	-0.1476	0.9999	0.9492	-0.0753	-0.2674	0.0702	D_driver
PCBD2	0.0457	0.1351	0.2450	0.0252	0.9999	1.1446	0.1949	0.3495	0.0265	up
POLG	0.0474	-0.0581	0.0383	-0.1545	0.9999	0.9435	-0.0838	-0.2840	0.0712	D_driver
POLRMT	0.0032	0.1025	0.1811	0.0238	0.9999	1.1079	0.1479	0.5069	-0.0664	D_driver

TARS2	0.0267	-0.0613	0.0165	-0.1391	0.9999	0.9405	-0.0884	-0.3305	0.0624	D_driver
TIMM22	0.0411	0.0273	0.1437	-0.0890	0.9999	1.0277	0.0394	-0.1321	0.1124	C_driver
UQCC1	0.0051	-0.0500	0.0532	-0.1531	0.9999	0.9513	-0.0721	0.0529	-0.1492	C_driver

**S3 sample**										
**primerid**	**Pr(>Chisq)**	**coef**	**ci.hi**	**ci.lo**	**fdr**	**fold_change[1]**	**log2FC[2]**	**β_D**	**β_C**	**category**
ABCD3	0.0356	-0.1285	0.0404	-0.2975	0.9025	0.8794	-0.1854	-0.0605	-0.1617	down
AIFM1	0.0288	0.1366	0.2956	-0.0224	0.8965	1.1464	0.1971	0.0635	0.1741	up
ATAD3A	0.0112	0.2397	0.4014	0.0780	0.8663	1.2709	0.3458	0.6220	0.0598	up
BCL2L13	0.0349	0.2164	0.3794	0.0534	0.9025	1.2416	0.3121	0.4376	0.1092	up
CLPP	0.0279	0.2283	0.4303	0.0263	0.8934	1.2564	0.3293	0.8299	0.0577	up
COA7	0.0241	0.1244	0.2559	-0.0072	0.8934	1.1324	0.1794	0.5235	-0.0750	D_driver
COQ2	0.0481	0.0982	0.2659	-0.0696	0.9025	1.1032	0.1416	0.4232	-0.1000	D_driver
COX7A2L	0.0260	-0.0187	0.2288	-0.2662	0.8934	0.9815	-0.0270	0.8782	-0.1770	C_driver
DTYMK	0.0379	0.3186	0.5820	0.0553	0.9025	1.3753	0.4597	0.6290	0.1328	up
DUS2	0.0435	-0.1062	0.0095	-0.2219	0.9025	0.8992	-0.1532	-0.4953	0.0567	D_driver
GCDH	0.0113	0.1882	0.3067	0.0697	0.8663	1.2070	0.2715	0.5581	0.0982	up
HIBADH	0.0304	0.0826	0.2493	-0.0840	0.9024	1.0861	0.1192	0.4467	-0.1055	D_driver
HSCB	0.0247	-0.1833	-0.0467	-0.3198	0.8934	0.8326	-0.2644	-0.4823	-0.0722	down
ISCA2	0.0344	0.2135	0.3822	0.0447	0.9025	1.2380	0.3080	0.3585	0.1472	up
MRPL12	0.0197	0.2203	0.4159	0.0247	0.8934	1.2464	0.3178	0.0785	0.2269	up
MT-ATP8	0.0346	0.0019	0.1920	-0.1882	0.9025	1.0019	0.0028	0.4119	-0.1496	D_driver
MTG1	0.0497	0.1795	0.3359	0.0231	0.9025	1.1966	0.2590	0.2880	0.1353	up
NTHL1	0.0367	0.0528	0.2121	-0.1066	0.9025	1.0542	0.0761	0.3427	-0.1329	D_driver
NUDT5	0.0451	0.1993	0.3910	0.0076	0.9025	1.2205	0.2875	0.9393	0.0598	up
OCIAD2	0.0189	0.1453	0.3512	-0.0606	0.8934	1.1564	0.2097	0.5217	-0.2081	D_driver
OPA1	0.0312	0.0639	0.2454	-0.1176	0.9025	1.0660	0.0922	0.5643	-0.1058	D_driver
OXR1	0.0457	-0.0326	0.1807	-0.2459	0.9025	0.9679	-0.0470	0.7170	-0.1550	C_driver
OXSM	0.0212	-0.1259	-0.0047	-0.2472	0.8934	0.8817	-0.1817	-0.5471	0.0579	D_driver
POLG	0.0055	-0.0562	0.0856	-0.1980	0.8646	0.9454	-0.0811	0.0857	-0.1810	C_driver
PRELID3B	0.0368	0.0192	0.1952	-0.1568	0.9025	1.0194	0.0277	-0.5528	0.1443	C_driver
PTCD3	0.0304	0.1533	0.3265	-0.0198	0.9024	1.1657	0.2212	0.6076	-0.0393	D_driver
RARS2	0.0388	0.0032	0.1799	-0.1736	0.9025	1.0032	0.0046	0.5194	-0.1270	D_driver
SIRT5	0.0319	-0.1232	-0.0014	-0.2450	0.9025	0.8841	-0.1777	-0.3243	-0.1170	down
SLC25A22	0.0434	0.0337	0.1587	-0.0912	0.9025	1.0343	0.0487	0.2987	-0.1431	D_driver
SLC25A38	0.0499	0.1323	0.2718	-0.0073	0.9025	1.1414	0.1908	0.4988	-0.0550	D_driver
SUCLG2	0.0229	0.0647	0.2699	-0.1404	0.8934	1.0669	0.0934	0.7952	-0.1108	D_driver
TOMM40	0.0263	0.2453	0.4489	0.0418	0.8934	1.2780	0.3539	0.6622	0.0491	up
TOP3A	0.0151	0.1598	0.2716	0.0480	0.8785	1.1733	0.2305	0.4770	0.1176	up

**S4 sample**										
**primerid**	**Pr(>Chisq)**	**coef**	**ci.hi**	**ci.lo**	**fdr**	**fold_change[1]**	**log2FC[2]**	**β_D**	**β_C**	**category**
BCL2L13	0.0404	-0.2169	0.1214	-0.5551	0.9917	0.8050	-0.3129	-0.0047	-0.3133	down
BPHL	0.0447	-0.0178	0.2097	-0.2452	0.9917	0.9824	-0.0256	0.2387	-0.2622	C_driver
CBR3	0.0171	-0.0952	0.2747	-0.4650	0.9917	0.9092	-0.1373	0.2842	-0.4284	C_driver
CBR4	0.0262	-0.3019	0.0088	-0.6125	0.9917	0.7394	-0.4355	-0.3574	-0.2744	down
FOXRED1	0.0252	-0.1432	0.1118	-0.3983	0.9917	0.8665	-0.2067	-0.1492	-0.2778	down

FXN	0.0464	-0.3518	-0.0658	-0.6379	0.9917	0.7034	-0.5076	-0.7376	-0.1709	down
GFER	0.0081	0.0277	0.4097	-0.3544	0.9909	1.0280	0.0399	0.6982	-0.3629	D_driver
LETM1	0.0090	0.3781	0.6164	0.1397	0.9909	1.4594	0.5454	0.6584	0.3125	up
METTL17	0.0024	-0.4097	-0.1740	-0.6454	0.9909	0.6639	-0.5910	-1.2847	-0.0601	down
MFN1	0.0203	0.0186	0.2719	-0.2347	0.9917	1.0188	0.0269	-0.6050	0.2588	C_driver
MMUT	0.0142	-0.3216	-0.0780	-0.5652	0.9909	0.7250	-0.4640	-1.1930	0.0223	D_driver
MRM3	0.0144	-0.2702	0.0847	-0.6250	0.9909	0.7632	-0.3898	-0.1160	-0.4338	down
MRPS11	0.0451	-0.4436	-0.0715	-0.8156	0.9917	0.6418	-0.6399	-0.5562	-0.3615	down
MRPS18A	0.0147	-0.3015	0.0742	-0.6771	0.9909	0.7397	-0.4349	0.1040	-0.4049	C_driver
MRPS31	0.0207	-0.4857	-0.1585	-0.8129	0.9917	0.6153	-0.7007	-1.2815	-0.2425	down
NDUFAF7	0.0406	-0.3194	-0.0614	-0.5774	0.9917	0.7266	-0.4608	-0.8312	-0.1080	down
NDUFS1	0.0366	-0.3959	-0.1171	-0.6747	0.9917	0.6731	-0.5712	-1.3419	-0.2146	down
NME6	0.0004	-0.3651	-0.1095	-0.6207	0.5579	0.6941	-0.5268	-0.9051	-0.2507	down
NUBPL	0.0261	-0.0089	0.1946	-0.2125	0.9917	0.9911	-0.0129	0.2902	-0.3299	C_driver
PMPCB	0.0403	-0.4159	-0.0893	-0.7424	0.9917	0.6598	-0.6000	-0.7036	-0.3166	down
QDPR	0.0052	0.0568	0.5389	-0.4253	0.9909	1.0584	0.0819	1.0651	-0.4038	D_driver
RHOT2	0.0244	-0.3251	-0.0235	-0.6268	0.9917	0.7224	-0.4691	-0.4639	-0.2638	down
RMDN3	0.0105	0.1285	0.4209	-0.1639	0.9909	1.1372	0.1854	0.7701	-0.2793	D_driver
SDHA	0.0406	-0.4190	-0.1054	-0.7326	0.9917	0.6577	-0.6045	-0.9334	-0.2434	down
SDHAF1	0.0334	-0.3545	-0.0878	-0.6211	0.9917	0.7016	-0.5114	-0.8946	-0.1084	down
SLC25A20	0.0399	-0.0119	0.2582	-0.2820	0.9917	0.9882	-0.0171	0.3000	-0.2501	C_driver
SLC25A28	0.0186	-0.3132	-0.0159	-0.6104	0.9917	0.7311	-0.4518	-0.4669	-0.2642	down
SLC25A38	0.0107	-0.2195	0.1537	-0.5928	0.9909	0.8029	-0.3167	0.0643	-0.5162	C_driver
SLC25A40	0.0232	-0.1021	0.1286	-0.3328	0.9917	0.9029	-0.1473	-0.0845	-0.2974	down
SYNJ2BP	0.0354	-0.2394	0.0875	-0.5663	0.9917	0.7871	-0.3453	-0.1377	-0.2957	down
TIMM22	0.0157	-0.4145	-0.0264	-0.8026	0.9917	0.6607	-0.5980	-0.2152	-0.4212	down

[1] fold-change = (exp(coef)). [2]log_2FC: base-2 log-fold-change (coef / log(2)).

Each row lists a gene (**primerid**) passing the nominal LRT p-value cutoff (Pr(>Chisq) < 0.05). **Pr(>Chisq)**: likelihood-ratio test p-value for the cond90-100% contrast; **coef** (log fold-change) and its 95 % confidence interval (**ci.hi, ci.lo**), representing the overall log-scale difference in expression; **fdr**: adjusted p-value (Benjamini–Hochberg) across all tested genes; **β_D**: hurdle-model discrete component coefficient, reflecting change in the fraction of cells expressing the gene; **β_C**: continuous component coefficient, reflecting change in per-cell transcript abundance among expressing cells.

In this table, each gene is also assigned to one of four regulatory categories based on the hurdle-model coefficients β_D, β_C, and the overall log-fold-change (coef): 1) up genes show both β_D and β_C positive (and coef > 0), meaning that not only do more cells turn the gene "on," but each of those cells also produces more transcript; 2)down genes have both β_D and β_C negative (and coef < 0), reflecting that fewer cells express the gene and that those cells express it at lower levels; 3) D_driver genes exhibit a significant change in β_D (matching the sign of the coef) while β_C remains near zero or opposite in sign. The regulation occurs predominantly through recruiting more (or fewer) cells to express the gene, with little change in how much each cell expresses. When coef > 0, more cells "switch on" the gene without boosting per-cell intensity; when coef < 0, fewer cells express the gene, again without major shifts in per-cell abundance. 4) C_driver genes show the converse pattern: β_C aligns with the sign of the coef, indicating a change in transcript level per expressing cell, while β_D stays near zero or opposite in sign. This reflects modulation of expression intensity within cells, rather than changes in the number of expressing cells.

Supplementary Table S5. MitoCarta3.0–annotated differentially expressed genes in pooled samples S1-S4 between high- and low-heteroplasmy (> 90 % vs. 0–10 %, LRT p < 0.05).

primerid	Pr(>Chisq)	coef	ci.hi	ci.lo	fdr	fold_change[1]	log2FC[2]	β_D	β_C	category
ABCD3	0.0036	-0.0621	0.0016	-0.1258	0.8330	0.9398	-0.0895	-0.0441	-0.0825	down
ACOT13	0.0428	0.0830	0.1569	0.0092	0.9426	1.0866	0.1198	0.2721	0.0106	up
AKR7A2	0.0036	0.0935	0.1565	0.0305	0.8330	1.0980	0.1349	-0.0851	0.1005	C_driver
CHCHD3	0.0247	-0.0722	-0.0133	-0.1312	0.9088	0.9303	-0.1042	-0.4286	-0.0387	down
COX7A1	0.0049	0.1265	0.2248	0.0283	0.8645	1.1349	0.1825	0.3064	-0.0033	D_driver
CYCS	0.0038	-0.1125	-0.0332	-0.1917	0.8482	0.8936	-0.1622	-0.5534	-0.0430	down
DNAJC15	0.0355	0.0038	0.0613	-0.0537	0.9269	1.0038	0.0055	-0.6287	0.0258	C_driver
EXOG	0.0363	-0.0788	-0.0161	-0.1414	0.9269	0.9243	-0.1136	-0.2288	-0.0077	down
FAM210B	0.0147	-0.1055	-0.0321	-0.1789	0.9088	0.8999	-0.1522	-0.3654	-0.0737	down
FARS2	0.0124	-0.0411	0.0314	-0.1135	0.9088	0.9598	-0.0592	0.1140	-0.0821	C_driver
GPX1	0.0095	-0.0574	0.0162	-0.1310	0.9088	0.9442	-0.0829	-0.4901	-0.0262	down
HSD17B4	0.0362	0.0034	0.0719	-0.0651	0.9269	1.0034	0.0049	-0.1722	0.0550	C_driver
MGST1	0.0436	0.0241	0.1490	-0.1008	0.9426	1.0244	0.0348	-0.2020	0.0907	C_driver
MICOS10	0.0034	-0.0746	-0.0159	-0.1333	0.8330	0.9281	-0.1076	-0.6297	-0.0410	down
MRPL55	0.0120	0.0866	0.1532	0.0199	0.9088	1.0904	0.1249	0.4041	0.0293	up
MRPL57	0.0212	0.0719	0.1276	0.0161	0.9088	1.0745	0.1037	-0.1048	0.0757	C_driver
MSRA	0.0145	-0.0787	-0.0075	-0.1499	0.9088	0.9243	-0.1135	-0.2950	0.0075	D_driver
MT-ATP8	0.0140	-0.0917	-0.0270	-0.1564	0.9088	0.9124	-0.1323	0.1335	-0.0936	C_driver
MT-ND5	0.0287	-0.0805	-0.0213	-0.1396	0.9088	0.9227	-0.1161	0.0000	-0.0805	down
MTG2	0.0397	0.0420	0.1022	-0.0182	0.9413	1.0429	0.0606	0.0425	0.0599	up
MTX3	0.0499	-0.0481	0.0124	-0.1086	0.9426	0.9531	-0.0694	-0.0609	-0.0685	down
MYO19	0.0422	-0.0488	0.0106	-0.1082	0.9426	0.9524	-0.0704	-0.0631	-0.0624	down
NDUFA6	0.0224	0.0101	0.0742	-0.0541	0.9088	1.0101	0.0145	-0.5727	0.0405	C_driver
NDUFA9	0.0449	0.0902	0.1613	0.0192	0.9426	1.0944	0.1302	0.2393	0.0252	up
NDUFAF7	0.0210	-0.0300	0.0178	-0.0778	0.9088	0.9705	-0.0433	-0.0353	-0.0655	down
NDUFV3	0.0268	-0.0680	0.0060	-0.1421	0.9088	0.9342	-0.0982	-0.0258	-0.0816	down
NOA1	0.0181	0.0518	0.1095	-0.0059	0.9088	1.0531	0.0747	0.0730	0.0655	up
OXSM	0.0113	-0.0384	-0.0033	-0.0735	0.9088	0.9623	-0.0554	-0.2752	0.0382	D_driver
PDE12	0.0315	-0.0353	0.0188	-0.0895	0.9222	0.9653	-0.0510	-0.0357	-0.0642	down
PDHX	0.0069	0.0251	0.0826	-0.0324	0.9088	1.0254	0.0362	0.1759	-0.0639	D_driver
PDK3	0.0121	0.0222	0.0691	-0.0247	0.9088	1.0224	0.0320	0.1864	-0.0721	D_driver
PPTC7	0.0260	-0.0680	-0.0072	-0.1289	0.9088	0.9342	-0.0981	-0.2355	0.0157	D_driver
SCO1	0.0266	0.0913	0.1594	0.0233	0.9088	1.0956	0.1318	0.2603	0.0197	up
SCO2	0.0470	0.0905	0.1636	0.0173	0.9426	1.0947	0.1305	0.2379	0.0366	up

SFXN1	0.0141	-0.0669	0.0010	-0.1349	0.9088	0.9353	-0.0965	-0.2474	0.0316	D_driver
SLC25A20	0.0148	-0.0499	-0.0058	-0.0940	0.9088	0.9513	-0.0720	-0.1496	-0.0553	down
SLC25A22	0.0407	0.0051	0.0320	-0.0218	0.9426	1.0051	0.0074	0.1150	-0.0674	D_driver
TARS2	0.0096	-0.0437	-0.0043	-0.0832	0.9088	0.9572	-0.0631	-0.2578	0.0334	D_driver
TIMM17B	0.0162	-0.0884	-0.0134	-0.1635	0.9088	0.9154	-0.1276	-0.3033	0.0047	D_driver
TRNT1	0.0059	-0.0467	0.0186	-0.1120	0.8856	0.9544	-0.0673	0.0250	-0.0812	C_driver
TXNRD2	0.0062	-0.0667	-0.0043	-0.1290	0.9088	0.9355	-0.0962	-0.0698	-0.0796	down
UQCC1	0.0015	-0.0699	-0.0085	-0.1314	0.7014	0.9325	-0.1009	-0.0567	-0.0975	down

[1] fold-change = (exp(coef)); [2]log_2FC: base-2 log-fold-change (coef / log(2)).

Supplementary Figure S1

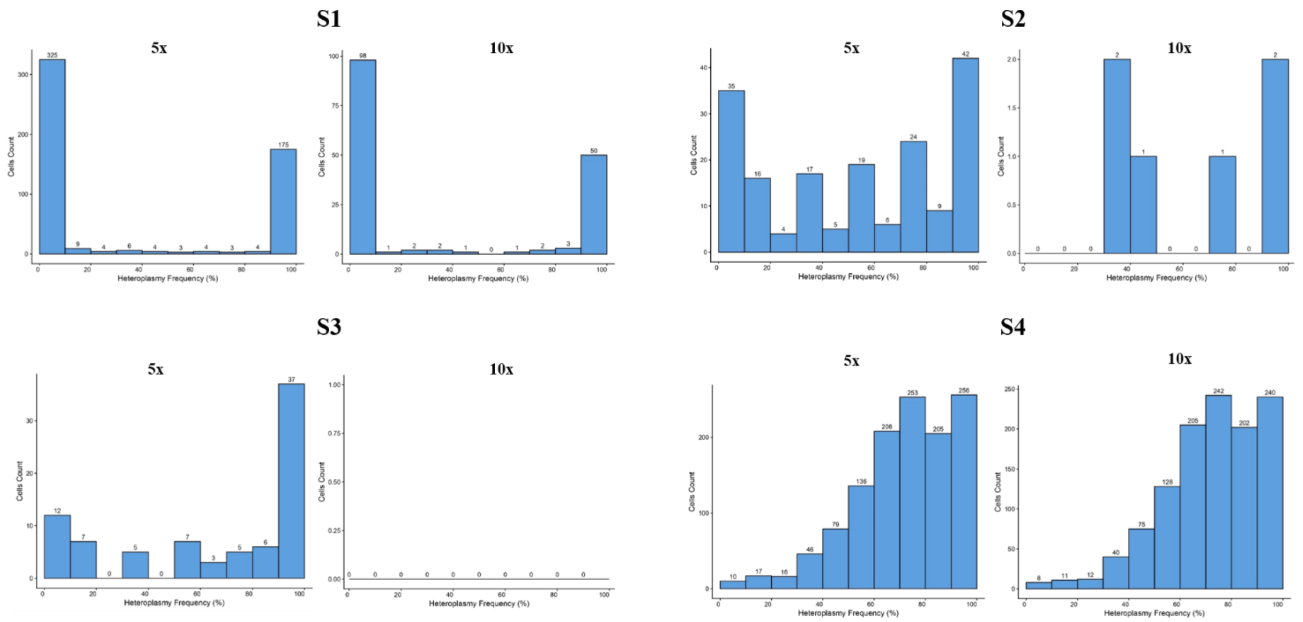

Figure S1. Distribution of per-cell heteroplasmy across four patient fibroblast samples, considering cells with ≥5 (5x) or 10 (10x) reads in the sample-specific mtDNA variant position.

The graphs showed the distribution of per-cell heteroplasmy in the samples S1-S4. The y-axis represents the number of cells, and the x-axis shows heteroplasmy frequency in percentage with 10% histo-gram bins.

Supplementary Figure S2

A **B**

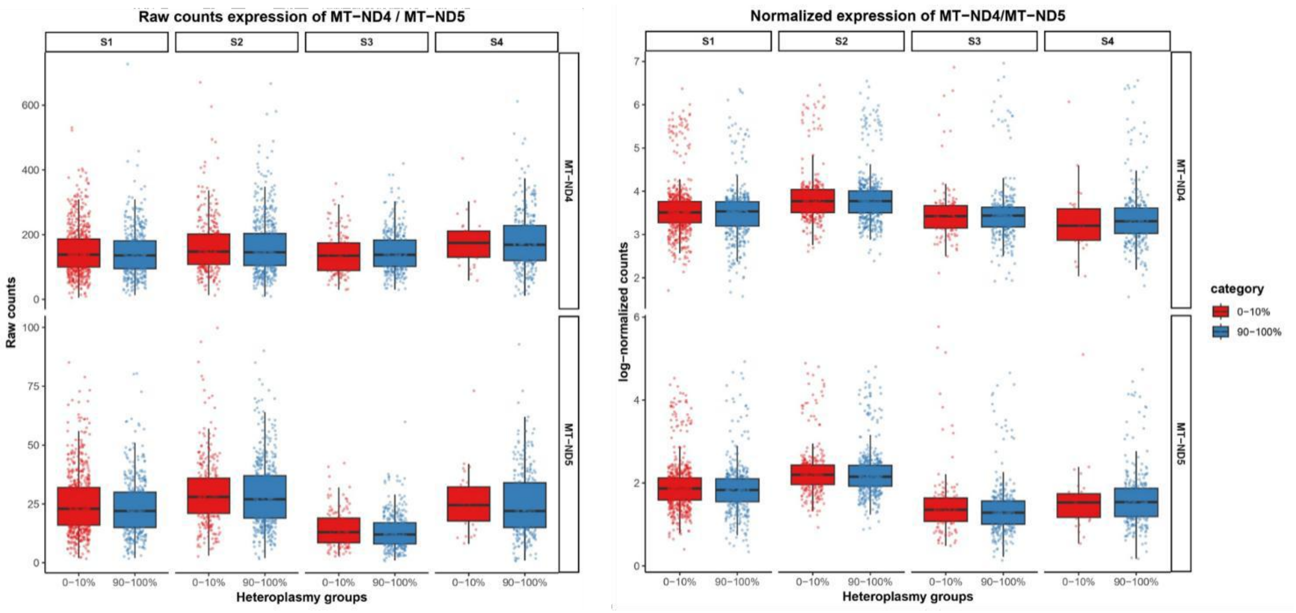

Figure S2. Raw and log-normalized expression of mitochondrial genes *MT-ND4* and *MT-ND5* stratified by heteroplasmy category (0–10 % vs. 90–100 %) across four patient fibroblast samples (S1–S4).

A) boxplots of raw UMI counts per cell, overlaid with individual data points; B) boxplots of log-normalized counts, overlaid with individual data points.

Supplementary Figure S3

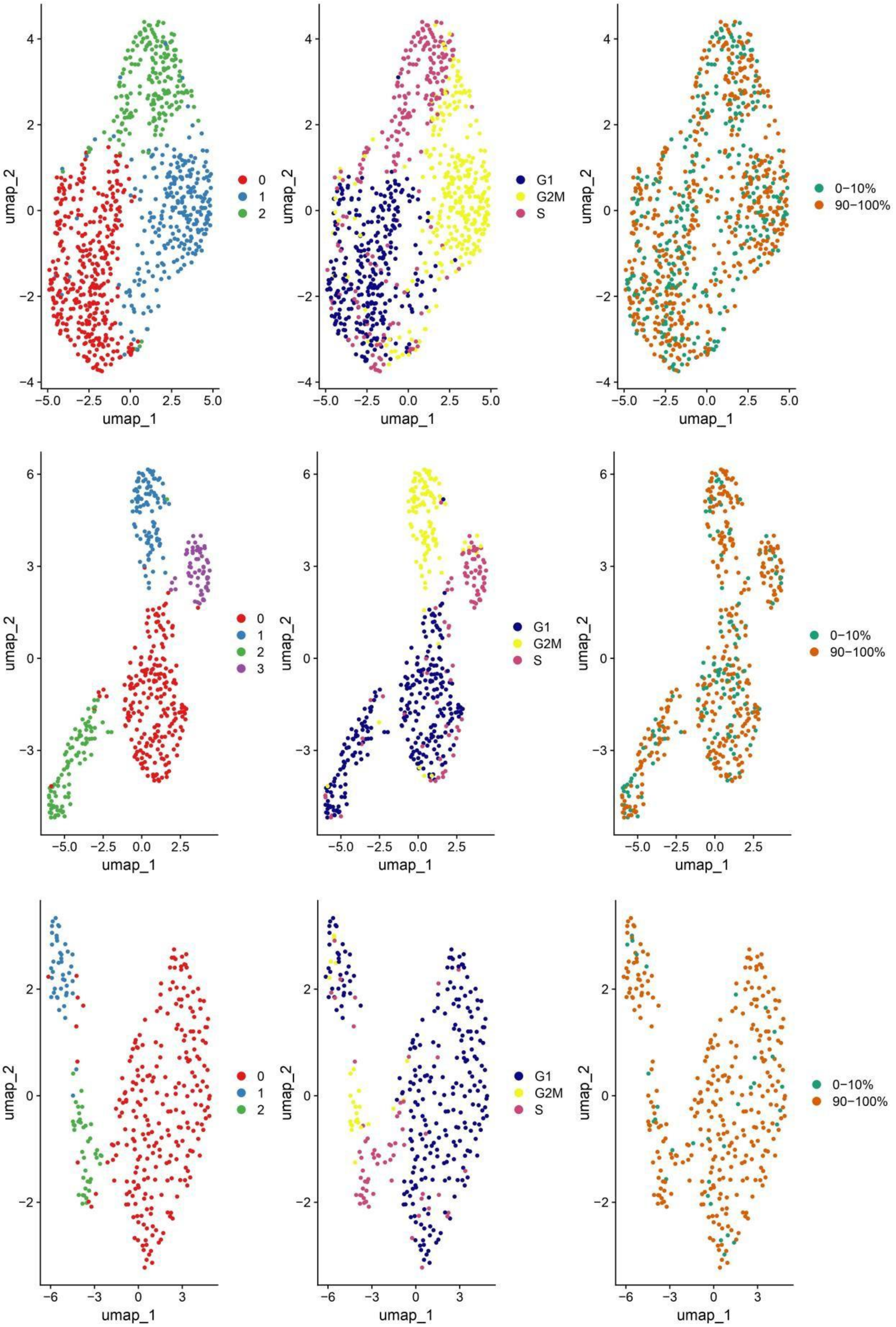

Figure S3. Per-sample UMAP embeddings for fibroblast lines S2–S4 (one row per sample)

Each rows shows one fibroblast line (S2-S3-S4); for each sample we display three side-by-side panels. Left panel: UMAP embedding (resolution 0.3; graph-based clustering) with cells colored by the cluster label to visualize the local transcriptomic structure. Central panel: the same embedding colored by cell-cycle Phase (G1, S, G2/M) assigned with CellCycleScoring. Right panel: the embedding colored by heteroplasmy class - low (0-10%) in teal and high (90-100%) in orange. Across all samples the two heteroplasmy classes are extensively intermixed within clusters, indicating that heteroplasmy level alone does not produce distinct global transcriptional clusters. By contrast, cluster composition by Phase is highly informative and generally mirrors the pattern observed in sample S1: clusters segregate into cell-cycle–related states (two G1-enriched clusters, an S-enriched cluster and a G2/M-enriched cluster). This interpretation is supported by multiple, independent lines of evidence (see main text and Supplementary Tables S1–S3): the Phase assignments from CellCycleScoring, the identity of cluster top markers (e.g., canonical S and G2/M genes) and preranked GSEA results (NES and adjusted p-values with leading-edge genes) all point to cell-cycle status as the principal driver of the UMAP structure. Note that the sharpness of cluster boundaries varies between samples, but in no case do high-heteroplasmy cells form a separate global transcriptional cluster.

Supplementary Figure S4

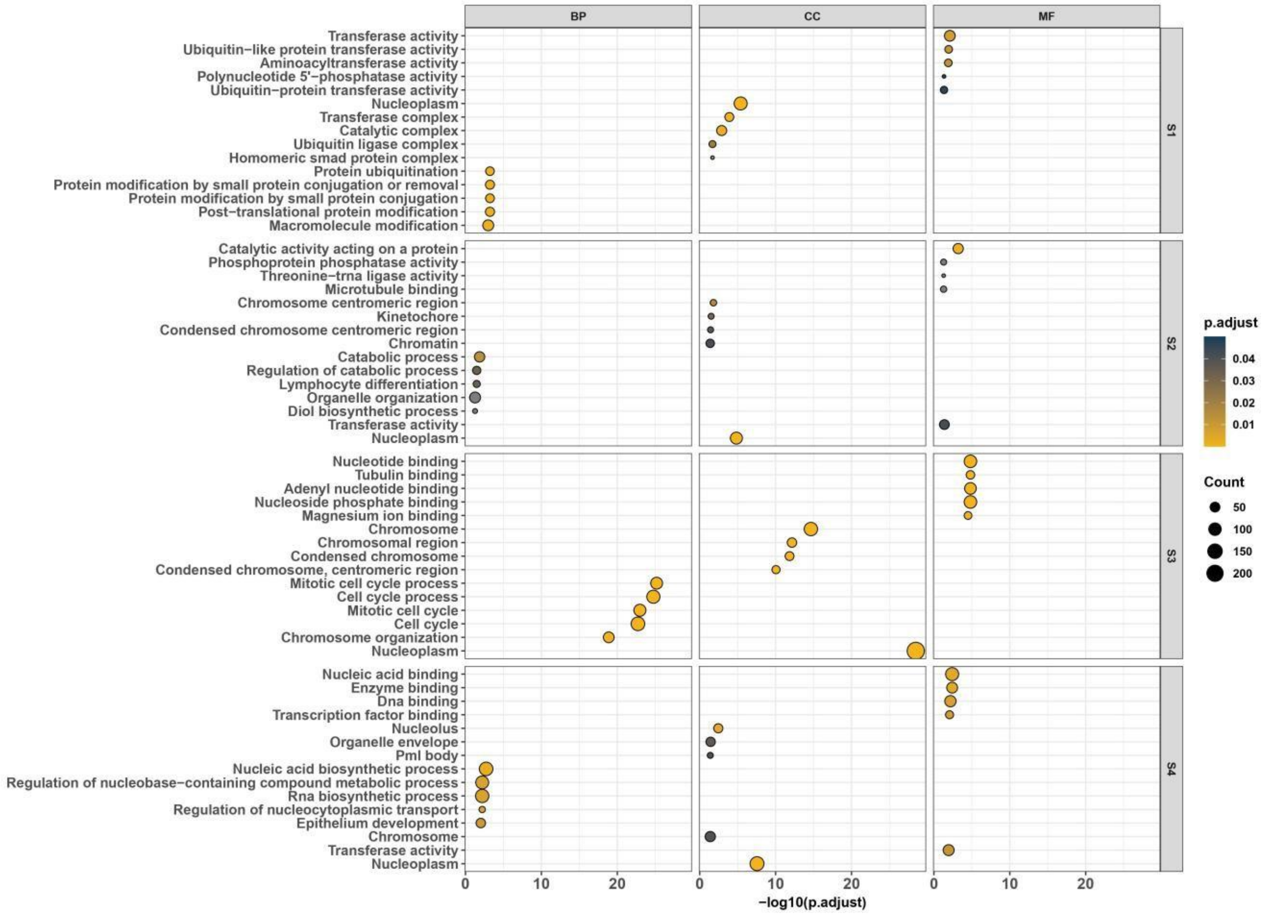

Figure S4. Ontology-stratified GO enrichment across four fibroblast lines (S1–S4) under extreme mitochondrial heteroplasmy (>90% vs 0–10%).

Gene Ontology (GO) enrichment results were stratified by ontology (Biological Process [BP], Cellular Component [CC], and Molecular Function [MF]) and filtered to retain the five most significant terms (ranked by adjusted p-value) for each ontology within each sample. Enrichment was performed using compareCluster with enrichGO (Benjamini–Hochberg correction; adjusted p-value threshold = 0.05), and the minimum gene set size was lowered to minGSSize = 2 to capture small but potentially relevant functional modules. Term descriptions were normalized (case formatting and trimming) to avoid duplication across panels. The plot displays −log10(adjusted p-value) on the x-axis and GO term descriptions on the y-axis. Dot size represents the number of differentially expressed genes (Count) contributing to each term, while fill color encodes the adjusted p-value (scale limited to 0 - 0.05). Faceting is organized by sample (rows) and ontology category (columns), with independent y-axis scaling per panel.
